# Insights into high-grade serous carcinoma pathobiology using three-dimensional culture model systems

**DOI:** 10.1186/s13048-023-01145-x

**Published:** 2023-04-11

**Authors:** Emily Tomas, Trevor G. Shepherd

**Affiliations:** 1 London Regional Cancer Program, The Mary & John Knight Translational Ovarian Cancer Research Unit, 790 Commissioners Rd. E. Room A4-836, London, ON N6A 4L6 Canada; 2 Department of Anatomy & Cell Biology, Western University, London, ON Canada; 3 Department of Obstetrics & Gynaecology, Western University, London, ON Canada; 4 Department of Oncology, Western University, London, ON Canada

**Keywords:** Epithelial ovarian cancer, High-grade serous ovarian cancer, 3D model systems, Spheroids, Patient-derived organoids

## Abstract

Epithelial ovarian cancer (EOC) research has become more complex as researchers try to fully understand the metastatic process. Especially as we delve into the concept of tumour dormancy, where cells transition between proliferative and dormant states to survive during disease progression. Thus, the in vitro models used to conduct this research need to reflect this vast biological complexity. The innovation behind the many three-dimensional (3D) spheroid models has been refined to easily generate reproducible spheroids so that we may understand the various molecular signaling changes of cells during metastasis and determine therapeutic efficacy of treatments. This ingenuity was then used to develop the 3D ex vivo patient-derived organoid model, as well as multiple co-culture model systems for EOC research. Although, researchers need to continue to push the boundaries of these current models for in vitro and even in vivo work in the future. In this review, we describe the 3D models already in use, where these models can be developed further and how we can use these models to gain the most knowledge on EOC pathogenesis and discover new targeted therapies.

## Background on epithelial ovarian cancer

Ovarian cancer is known as the most lethal gynaecological cancer in the developed world, with three main subtypes namely epithelial, germ cell, and stromal cell [1, 2]. However, most patients are diagnosed with epithelial ovarian cancer (EOC), which again can be categorized into different histotypes including high-grade serous, low-grade serous, mucinous, clear cell and endometrioid. With a lack of distinguishing symptoms and predictive biomarkers, patients are usually diagnosed at FIGO stage III or IV and commonly with the most aggressive histotype, high-grade serous carcinoma (HGSC).

**Fig. 1:**
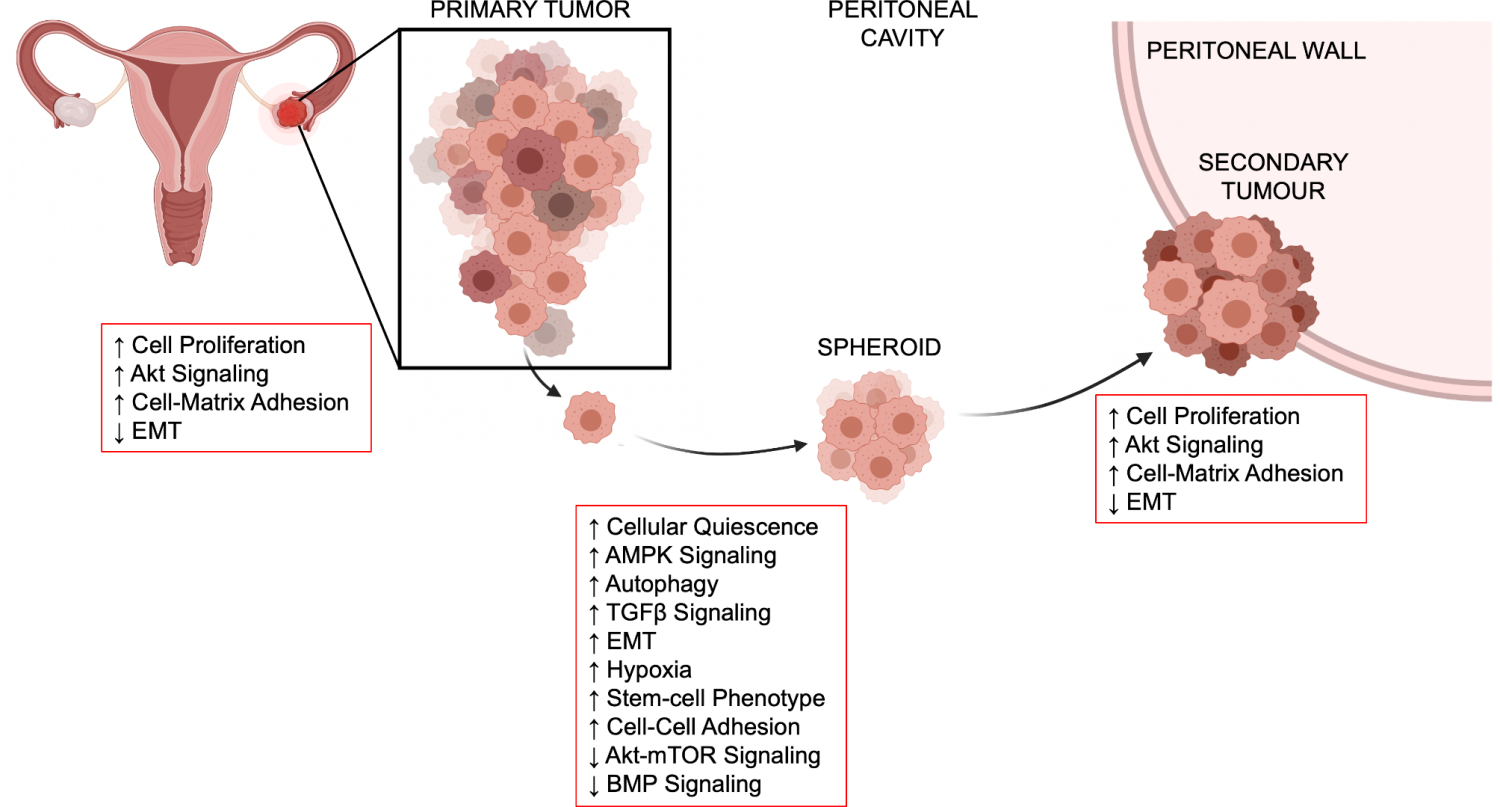
**Pathobiology of EOC cells within tumours and spheroids throughout disease progression.** This schematic demonstrates the complex nature of EOC cells where they undergo reversible cellular and molecular changes during metastasis, specifically highlighting dormant-to-proliferative switching. Cells will break off from tumours and disseminate into the peritoneal cavity, within the malignant ascites fluid, to form multicellular aggregates called spheroids. As cells cluster in suspension, they alter their intracellular signaling pathways (e.g. proliferation, adhesion, EMT) to become dormant and often drug-resistant to evade cell death. These changes are also reversible in the sense that spheroids can reattach to peritoneal surfaces and switch their biology back to a tumour-like state in order to grow secondary lesions. Created with Biorender.com.

Another reason for this lack of symptoms and quick progression is due to the unique development of metastatic disease. EOC develops from serous tubal intraepithelial carcinoma (STIC) lesions in the fallopian tube epithelium (FTE), due to mutations in *TP53*, which advances into an invasive carcinoma tumour [3, 4]. Then, cancerous cells can detach from tumours and disseminate within the peritoneal cavity forming these multicellular clusters called spheroids [4]. Cells within spheroids adapt to become dormant and drug resistant, allowing them to evade treatments and survive independently. This involves changing the regulation of their intra- and intercellular signaling pathways through a concept known as tumour dormancy. Some examples are exhibited in Fig. 1, such as cellular quiescence, autophagy, cell adhesion, hypoxia, epithelial-mesenchymal transition (EMT), and bioenergetic stress [5–17]. Malignant ascites fluid tends to build up within this same cavity and allows for easy transit of spheroids to form potential secondary tumours [18]. In turn, this combination of cellular dormancy and spreading through spheroids allows for an aggressive cancer to develop mostly undetected.

Due to this complexity, it is unlikely that a single treatment would be effective for all EOC patients. The standard regimen for advanced patients is to undergo peritoneal debulking surgery with pre- or post-surgical chemotherapy treatments, often a combination of carboplatin and paclitaxel [1]. These two chemotherapeutic agents inhibit cancerous cells that are actively dividing; thus, they are much less effective against dormant cells. Other more targeted therapies could be used, including PARP-inhibitors (PARPi) for BRCA1/2 mutation carriers and the anti-angiogenic bevacizumab as maintenance therapy, but these are only for a select few patients who meet the treatment criteria [18–21]. Overall, this creates a clinical dilemma for HGSC patients where they have an aggressive cancer with limited treatment options.

To understand this disease and how best to treat it, researchers use a variety of different cell culture systems with three-dimensional (3D) models being the most popular to accurately portray this disease. The most heavily used approach for EOC utilizes the in vitro suspension spheroid model system to observe its metastatic pathway, although there is increasing interest in other 3D models that could prove useful. For example, tumour initiation and organization can be observed through patient-derived organoid models, or the tumour microenvironment (TME) and cell-cell interactions could be observed through ex vivo patient-derived co-culture models with the respective cancer-associated cell types [22]. Here, we review the various 3D models already being utilized for EOC research, how they allow us to further our understanding of this devastating disease and how we can improve on these models to advance research in this field.

## Tumour dormancy in EOC

Based on decades of EOC research, multiple signaling pathways have been implicated in a process called tumour dormancy. This occurs when cells become dormant to survive during metastasis as spheroids and then revert their biology to a proliferative state for reattachment and secondary tumour growth [23, 24]. As such, dormancy cannot be observed within 2D culture systems, in which cells adhere to stiff tissue culture plastic promoting proliferation, but it has only been observed through suspension culture 3D spheroid model systems. As well, these intracellular signaling changes are often intertwined via cross-regulation thus promoting steps of EOC metastasis in additional ways. Identifying the key proteins and their specific interactions within these unique metastatic cells has been critical in understanding advanced disease and discovering new therapeutic avenues.

*AKT signaling*. The PI3K-Akt-mTOR pathway is one of the most widely mutated and activated signaling pathways in human cancers, since it has the capacity to promote cell proliferation, protein translation, cell motility and migration, and enhance epithelial-mesenchymal transition, to name just a few [25]. Mutations are observed in this pathway in human EOC, with activating missense mutations in *PIK3CA*, loss of function deletions in *PTEN*, and amplifications in *AKT1*, *AKT2*, and *AKT3*, among the different histotypes of this disease [26, 27]. Our results however showed reduced phosphorylation of Akt at serine 473 in HGSC cell lines and concomitant downstream signaling effects of lower phospho-p70S6K and phospho-4EBP1 [25, 28]. Initially, elevated Akt activity was expected to promote EOC cell survival while in suspension to block anoikis induction. However, we now know that this downregulation in Akt-mTOR signalling drives at least two key phenomena in EOC spheroids: cellular quiescence and autophagy.

*Cellular quiescence*. This process is very important for dormancy and has been extensively observed in EOC spheroids. It is also involved with increased resistance to cytotoxic chemotherapy, thus supporting the important role that these multicellular clusters may play in disease progression [29]. As described above, downregulation of Akt activity leads to the concomitant decrease in Skp2, with increased cyclin-dependent kinase inhibitor p27 and Rb1 related protein p130 expression. This result was recapitulated in proliferating adherent HGSC cells that were directly treated with the Akt inhibitor Akti-1/2, leading to arrest of cells in the G0/G1 phase of the cell cycle [25, 28]. Interestingly, upon the reattachment of spheroids, cell proliferation is resumed in cells that are emanating from the attached spheroid. This process requires the reactivation of Akt, and we have coined this process the dormant-to-proliferative switch as observed in tumour dormancy systems for other human cancers.

*Autophagy*. Macroautophagy, otherwise termed autophagy, is a general intracellular degradation process of organelles, macromolecules and in some cases pathogens [17]. Typically, autophagy is a universal stress-induced phenomenon under nutrient-depleted and starvation-like conditions to facilitate the generation of alternative substrates for energy production. Autophagy has tumour-suppressive activity in early malignant states to drive senescence as well as cell death. However, it is regarded as a key mechanism that can promote cancer cell survival under hypoxia in avascular tumours, nutrient depletion in rapidly growing tumours, or in the face of chemotherapy [30]. Indeed, our group observed that autophagy is rapidly induced in EOC spheroids, and this is driven by two parallel signaling pathways working in opposite orientations: downregulation of Akt-mTOR and upregulation of AMPK [10, 17]. Interestingly, we also determined that the canonical Beclin-1 mechanism for autophagy activation is not required in EOC spheroids, however ULK1 within the autophagy initiation complex (AIC) is activated and required in EOC spheroids [17, 28, 30]. Autophagy is necessary to promote cell survival while in suspension in spheroids, and it may also be implicated in promoting chemotherapy resistance in these structures [10]. It has also been implicated in mediating ovarian tumour growth and establishment of metastasis, perhaps through the downregulation of the tumour suppressor ARHI. Overall, these results strongly support autophagy is necessary to maintain EOC cell viability under a dormant-like state.

*TGFβ and BMP signaling.* The transforming growth factor-beta (TGFβ) superfamily of cytokines has widespread implications in human cancers. With a focused description of its role in EOC spheroids, we have observed the reciprocal expression and activity between bone morphogenic protein (BMP) and TGFβ signalling, with the former being decreased in spheroids and the latter being increased [31]. This alternating signaling activity is required for efficient spheroid formation, and most importantly to control their reprogramming of EMT. This is characterized by the canonical downregulation of E-cadherin and upregulation of transcription factors Snail, Slug, Twist, and ZEB1/2 [13]. Again, like the dormant-to-proliferative switch mediated by Akt, TGFβ signalling activity and this EMT phenotype are reversible upon spheroid reattachment. This is a process that can partly explain how metastatic EOC cells will possess epithelial marker expression in both primary and secondary tumours, but more mesenchymal markers in spheroids [32]. We propose that this plasticity is crucial for efficient spread and establishment of secondary lesions in the unique peritoneal environment of advanced EOC.

*LKB1 signaling*. EOC cells trigger intracellular stress response signaling when they detach into suspension, as well as being deprived of growth factors, extracellular matrix (ECM) components, nutrients, and oxygen [33, 34]. A key pathway that monitors these stressors in mammalian cells is the LKB1-AMPK signaling pathway. We observed that EOC cells have a characteristic decrease in intracellular mitochondrial redox potential and ATP levels [35]. AMPK senses the increased AMP/ADP:ATP ratio leading to its phosphorylation at T172, followed by metabolic reprogramming of cancer cells by shifting away from anabolic metabolism to a more catabolic phenotype [36]. This is crucial to yield new substrates for energy production in cancer cells that are energy- and substrate-depleted. We observed that both LKB1 and AMPK are increased in phosphorylation, expression, and signaling activity [35]. Interestingly, however, LKB1 is not required for AMPK phosphorylation and activity. Instead, CAMKKβ is required to phosphorylate AMPK at T172, and we have recently observed that CAMKKβ-AMPK signaling is required for induction of autophagy in EOC spheroids [10, 37]. To further our understanding of LKB1 activity requirements in EOC spheroids we performed a multiplex-inhibitor beads-mass spectrometry approach to discover other kinases that may be impacted by LKB1 loss using OVCAR8 cells with *STK11* deleted by CRISPR. We identified that the AMPK-related kinase NUAK1 is significantly reduced in phosphorylation and expression levels due to LKB1 loss in EOC spheroids [38]. Both LKB1 and NUAK1 are required for spheroid formation and more importantly for efficient metastatic potential in mouse xenografts. Without proper activity and function of this pathway, EOC cells under these stressors will have reduced viability and metastatic capacity.

*Cell adhesion*. In order for EOC disease progression, cells require both cell-cell and cell-matrix interactions for efficient spheroid formation and tumour growth, respectively. We found that NUAK1 performs this function since its loss impairs cell adhesion under both adherent and spheroid conditions [38]. In EOC cells and spheroids, NUAK1 controls the expression of important adhesion molecules and ECM substrates, such as fibronectin and beta-integrins. Gong et al. determined that both fibronectin and integrin receptors are responsible for adhesion in turn promoting invasion and metastasis [39]. Another group discovered that versican, a secreted proteoglycan protein found in the stroma, promotes EOC tumour metastasis and increased invasiveness [5]. Both groups highlighted the importance of understanding spheroid biology to identify new therapeutic targets, such as fibronectin and versican.

*Stem-cell like phenotype*. The ability of EOC cells to maintain stem-cell like properties has been implicated in disease recurrence and chemotherapy resistance. Cancer stem cell (CSC) populations, like those in other cancer types, facilitate EOC cells to become immunosuppressive, invasive, and platinum-resistant [40, 41]. More effective therapeutic targeting of these niche populations could prove useful to prolong disease-free and overall survival in patients. Common CSC markers include CD133 surface antigen expression and high ALDH1 enzymatic activity; however, more research into the specific EOC CSC markers and their implications in spheroids would be beneficial [40, 42]. Researchers could evaluate the proportion of CSCs within a tumour and spheroid structure, design markers specific for EOC, and determine how they affect EOC disease progression. For example, patient ascites fluid samples have been be used to study CSC populations and how they may contribute to chemotherapy resistance in patients [43–45].

*Hypoxia.* It is well-known that hypoxic cores exist within tumours and spheroids where cells are oxygen- and nutrient-deprived. Significant metabolic reprogramming allows cancer cells to survive in this state within these large structures. We found significant levels of necrosis and hypoxia in EOC intraperitoneal xenografts lacking LKB1-NUAK1 expression, implicating that this pathway may be required as a key hypoxic response during EOC metastasis [37]. Notably, hypoxia can lead to several other intracellular changes that sustain chemotherapy resistance and other malignant properties integral for metastasis [14, 46].

## In vitro spheroid culture

**Fig. 2:**
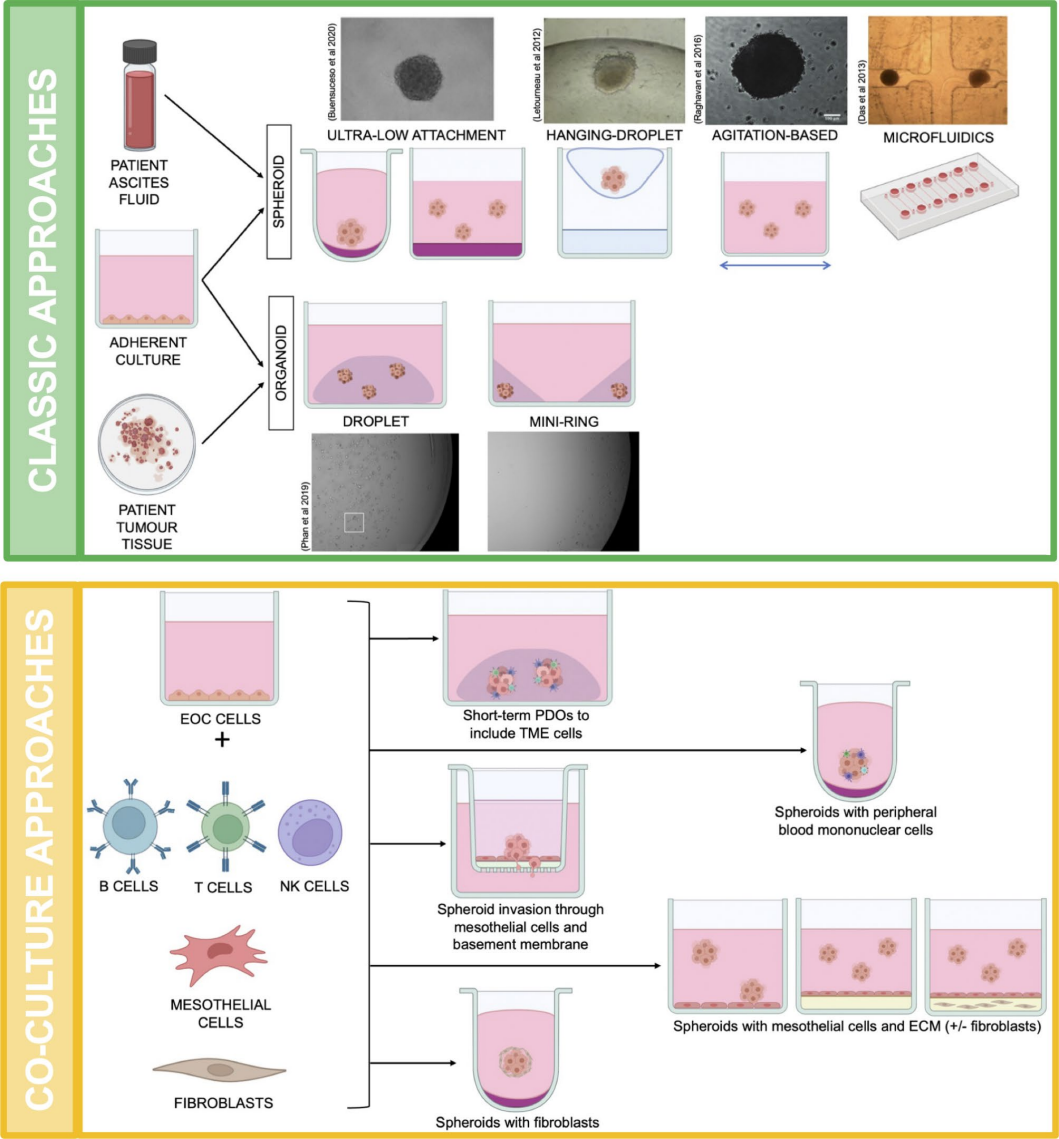
**Methodology behind the various spheroid and organoid 3D model systems for EOC research.** As demonstrated in the first green panel, ovarian cancer cell lines maintained on adherent cultures or fresh patient samples can be used to generate spheroids or organoids. There are four main spheroid models including ultra-low attachment plates, hanging-droplets, agitation-based and microfluidics. Organoids can be generated as a droplet or mini-ring structure of Matrigel? or Cultrex BME?. Utilizing these classic spheroid and organoid approaches, another avenue to study EOC in a 3D capacity is through co-culture or organotypic model systems as shown in the second yellow panel. These models require multiple cell types, thus, EOC cells can be cultured with other cells associated with tumours or spheroids, such as the common immune cells found within the TME and any surrounding peritoneal cells like fibroblasts and mesothelial cells. This allows researchers to observe and differentiate the importance of these complex interactions within tumours and spheroids. Created with Biorender.com.

The use of in vitro spheroids has been integral in understanding how EOC metastasizes within patients, the biology behind metastatic spheroids and how to potentially exploit this process for more treatment options. Several groups have clearly demonstrated the importance of studying 3D spheroids with the many discoveries of altered pathobiology outlined in the above section. There are multiple ways to culture spheroids in vitro, as shown in Fig. 2, and each one has their own advantages in current research.

Most of our work on spheroids focuses on the natural aggregation of cells in suspension using ultra-low attachment (ULA) cell culture plastic, usually in round-bottom plates for easily reproducible spheroid formation [37]. However, there is a similar approach where a hanging droplet is placed on the underside of the plate lid which allows gravity to cluster the cells into spheroids, again making them easily reproducible for any downstream analyses [45, 46]. Allowing spheroids to form in round-bottom ULA plates greatly facilitates numerous other assays, namely: cell viability assays, transferring spheroids, performing drug treatments and virus infections, co-culturing with additional cell types, and long-term culture since medium changes can be done with very minimal disruption of spheroids within each well. Another classic spheroid model uses an agitation-based approach where EOC cells are seeded onto plates that have agarose on the bottom of the culture vessel and incubated with constant agitation [44]; however, this technique exposes EOC cells to additional shear forces that may affect cell biology and viability.

These various in vitro spheroid model systems allow for researchers to observe tumour dormancy and any changes in cellular behaviour that occur. In addition, direct analysis of patient-derived spheroids can be done where malignant cells collected from patient ascites are cultured using these different platforms. With advanced disease being so common in EOC, there is a clear advantage to directly compare these spheroids to tumour structures within the same patient. Even though there has been evidence that EOC spheroids have a phenotype similar to tumours, more research comparing these two states needs to be done to fully understand the concept of tumour dormancy and reprogramming events as they relate to EOC disease progression [4].

## Ex vivo 3D culture models

A technical hurdle in 3D cell culture systems to study the biology of ovarian tumour cell clusters is the ability to visualize and monitor these structures in real-time. This challenge has been met by customized microfluidics chambers that facilitate microscopic evaluation of microdissected tumours or spheroids which can be held in place within chambers [14, 47–49] (Fig. 2). For example, St-Georges-Robillard and colleagues (2019) applied microfluidics “on-chip” analysis to overcome limitations of assessing cells within spheroids using techniques that may be destructive (e.g., single cell or FACS analysis), or provide insufficient resolution (i.e., confocal microscopy). To this end, they applied fluorescence wide-field hyperspectral imaging using two different fluorophores and mixed cell populations in co-cultured spheroids in microfluidic chips. Their technique allowed the assessment of thousands of trapped spheroids in a single step while exposed to external stimuli. The specialized fluorescence microscopy used in this study sought to overcome several limitations with visualizing living cells within a spheroid context, namely: resolution, loss of signal, requirement of optical clearing, overlap of fluorescence signals, and shorter working distances. This system was rapid, precise, and versatile since multiple fluorophores could be used.

Marimuthu and colleagues (2018) addressed another issue that clusters of differing sizes will have very different underlying biological responses to stimuli, such as hypoxia [47]. To address this, they developed and applied an integrated platform to form, treat, stain, and image multi-sized spheroids using microfluidic funnels and single inlet multi-size spheroid (SIMSS) chips. A hydrophobic surface facilitated long-term device storage and they assessed several aspects of fluid system, including filling dynamics, droplet and trapping stability, and shear stress. Wider applicability within laboratories is possible since their chips are compatible with a 96-well cluster plate format and spheroids can be transferred using simple contact transfer. This platform also allows for easy application of patient-derived spheroids or microdissected tumours as defined by the Mes-Masson group. In turn, analysis of patient-derived samples for effective drug screening using spheroid cultures becomes seamless, as demonstrated with their comparison among 3D spheroid model systems to measure carboplatin efficacy [49].

## Patient-derived Organoids of EOC

The ability to culture ovarian tumours ex vivo has rapidly emerged as a crucial 3D model system for research. Organoid model systems incorporate multiple cell types to represent the tissue from which it’s derived, or in a cancer context, into its representative tumour type [22, 50]. Over the past 20 years, cancer biologists have evolved this organoid model system for testing therapeutics in a high-throughput manner while capturing interpatient and intratumoral heterogeneity in many different cancer types.

Multiple groups have accomplished this for EOC with systematic testing of special growth media conditions to facilitate the viability of tumour cells from biopsy material and malignant ascites fluid. Microdissected tumour cells are dissociated into single cell suspensions or small clusters, then seeded within a basement membrane extract such as growth factor reduced Matrigel® or Cultrex BME® at defined cell concentrations [51, 52]. The versatility of this matrix was demonstrated by the Soragni group, which developed a new mini-ring organoid culture approach as opposed to the traditional dropseed approach that forms a solid dome per well [53] (Fig. 2). This allowed their patient-derived organoid development and drug treatment protocols to be automated and less labor intensive, while still recapitulating EOC patient tumour histotype and morphology.

Matrix-bound EOC organoids are overlaid with a defined cell culture medium, oftentimes using two-to-four different combinations of supplements. This is done since at the time of initial ex vivo PDO culture, it is unknown what factors may be required for optimal growth for each individual EOC tumour sample. However, several factors are commonly used to promote EOC organoid propagation, such as B27 supplement, nicotinamide, EGF, and inhibitors to ROCK and TGFβ type I receptors [51]. Other studies have looked more closely into these components for efficient EOC organoid growth based on histotype. According to Hoffman et al. (2020), activation of Wnt signalling can be detrimental for HGSC PDOs, whereas activation of BMP signalling with the addition of BMP2 and removal of the antagonist Noggin from growth media can promote long-term growth of these organoids [54]. More recently, addition of the HER3 ligand, neuregulin, has also been shown to enhance the long-term passage of PDOs while still maintaining integrity of the HGSC phenotype as determined by standard immunohistochemistry markers [55]. Of course, there still exists a need to determine the optimum media components for the other EOC histotypes, as well.

Experiments can be performed on early-passage PDOs, with the idea that ex vivo tumour PDOs offer a rapid evaluation of sensitivity to therapeutics in parallel with a patient’s on-going treatment protocol (i.e., PDO avatar). This was best exemplified by the Hill et al. study in which short-term cultures of HGSC PDOs were assessed for homologous recombination DNA repair deficiency and sensitivity to PARPi [56]. However, the ability to expand PDOs to later passages facilitates a greater capacity for therapeutics testing, reproducibility of experiments, and the cryopreservation of characterized and validated PDOs for specific histotypes. Indeed, several studies have confirmed that late-passage EOC PDOs maintain the original histotype, and more importantly the genomic complexity and transcriptome relative to the original tumour and early passage PDOs. However, a much smaller proportion of EOC samples survive to reach a point to be considered a successful late-passage PDO (> passage 5). In support of this idea, De Witte et al. highlighted the importance of developing stable PDOs from multiple tumour sites to get a comprehensive understanding of metastatic disease, genomic variability and high-throughput drug screening [52]. Another utility of well-established late-passage PDOs is the ability to implant into immune-compromised mice to establish PDO xenografts (PDO-X) [49, 57]. This is useful to validate drug sensitivity results observed in culture with potential efficacy in a pre-clinical model organism.

To gain a full understanding of ovarian cancer initiation and progression, some researchers have established FTE organoids that recapitulate the functional unit of the tubal epithelium with distinct polarization of both secretory and ciliated cells. One group developed FTE organoids through forced differentiation of iPSCs into progenitor FTE cells using niche transcription factors [58, 59]. A more direct approach can be done by isolating cells from normal distal fallopian tube tissue of non-cancer patients to culture in an organoid model system [60]. Likewise, others have demonstrated the ability to establish mouse oviduct and ovarian surface epithelial (OSE) organoids [61]. For example, the Clevers group used mouse oviduct organoids for lentivirus-mediated CRISPR ablation of *Trp53* to understand its genetic loss in HGSC development [62]. FTE organoids require their own set of media components to sustain growth in culture. Kessler and colleagues compared culture conditions between FTE and HGSC PDOs. They established that normal human FTE PDOs require BMP signaling inhibition via Noggin and Wnt activation via R-spondin and Wnt3a; however, the opposite scenario is required for successful propagation of HGSC PDOs [54, 63]. The ability to grow and genetically manipulate FTE organoids will prove very useful as a control model for testing novel therapeutics against HGSC. In addition, they can be utilized for further investigation into the additional mutational events and pathobiological changes required to drive precursor STIC lesions into ultimate HGSC malignancy.

PDOs can be used for a multitude of different techniques to gain new insights into EOC pathobiology. First and foremost, it is imperative to perform immunohistochemical stains of fixed and embedded PDOs using defined markers to confirm specific histotypes, e.g., PAX8 and p53 for HGSC, HNF1B for clear cell carcinoma, ER and PR for endometrioid [51]. The use of single-cell RNA sequencing and flow cytometry can be applied to better comprehend the vast inter- and intrapatient heterogeneity seen in both cancer cells and the microenvironmental components of tumours. This heterogeneity highlights the transcriptomic differences seen in tumours and the interactions with the microenvironment, which may impact the variable therapeutic efficacies in patients. Comprehensive morphological analysis of PDO growth to determine underlying metabolic activity of cells can be achieved. For example, Nelson et al. demonstrated mitotic heterogeneity utilizing both broad and single-cell analyses of PDOs [64]. In addition, there have been developments into 3D live cell fluorescence-based assays for a more in-depth investigation into organoid structures and transcriptomic differences as demonstrated by Kim and colleagues, but more optimization and improved versatility need to be resolved [65]. The biggest limitation of organoids is the increased labor and resources required for culture, unlike spheroids which can be quickly and easily developed using more standard culture conditions. As PDO protocols become more developed, and perhaps as the costs for reagents are reduced, this issue may be alleviated.

Utilizing FTE organoids and tumour PDOs will allow researchers to gain a better understanding of tumour growth using this representative 3D model system. Indeed, organoids have almost the same capacity as spheroids for efficient molecular analyses. Thus, a direct comparison between spheroids and organoids may highlight variations in drug efficacy between these models, and also prove useful when investigating dormant-to-proliferative switching to determine new and unique therapeutic strategies for advanced EOC.

## Co-culture model systems

Direct collection of multicellular clusters from malignant ascites and even short-term ex vivo cultures can be useful for assessing both tumour and non-tumour cell types residing within these 3D structures. This can be used to validate whether observations made with in vitro generated spheroids or organoids are recapitulated in native clusters from the ascites or tumours. Groups have established both spheroid and organoid co-cultures in which ovarian cancer cells are combined with mesothelial cells, fibroblasts, or immune cells to investigate heterotypic cell interactions and their impact on tumour biology (Fig. 2).

The primary barrier at most sites of EOC metastasis within the peritoneal cavity is the mesothelium. As such, assays have been developed to visualize and measure the ability of EOC cells and spheroids to attach, displace and invade through mesothelial surfaces. This is best demonstrated by Iwanicki and colleagues where they elucidated the mechanisms used by EOC spheroids to attach and spread through mesothelial cell monolayers using non-transformed LP9 human mesothelial cells [66]. They demonstrated that EOC cells within spheroids induce integrin- and myosin-generated forces to displace mesothelial cells for penetration and invasion. A similar system was used to assay for proteins present at the leading edge of the invasive front of EOC spheroids pushing through the mesothelium [67]. This confirmed the important interaction between cancer cells and mesothelial cells for their adhesive and invasive properties during EOC progression.

One of the most common sites of EOC metastasis in the peritoneal cavity is the omentum, which is a highly heterogenous tissue composed of adipocytes, connective tissue, blood vessels and lymphatics to control infection, inflammation, and damage within the peritoneal cavity [68]. Many reports have identified key factors in the omentum that can either attract metastatic EOC cells to this site or promote the adhesion and growth of secondary tumours [69]. Development and application of a co-culture system that mimics this tissue can be extremely useful to understand the molecular and cellular interplay involved in metastasis, as well as assay for therapeutics to block this process. Indeed, the pioneer in this area has been the Lengyel group where they isolated normal omentum from healthy patients to study the interplay of fibroblasts, mesothelial cells, and specific ECM components with EOC cells. They established a layered co-culture system using transwells in which fibroblasts embedded in ECM were laid down first, followed by a monolayer of mesothelial cells [70]. EOC cells were seeded into the transwell chamber to assess adhesion, invasion, and migration through this organotypic omentum co-culture model (Fig. 2). This powerful and unique co-culture model was utilized as a powerful and efficient therapeutics screening platform to identify novel compounds that block EOC cell adhesion and invasion [71]. Similarly, the Balkwill group established various tri-, tetra- and penta-cultures, also utilizing patient omental samples to isolate fibroblasts, adipocytes, and monocytes, to study the influence of omental components on HGSC cells and the deposition of ECM [72–75]. Using these patient-derived co-culture systems, both groups were able to faithfully recapitulate early and late steps of invasion through histological comparisons of their in vitro co-culture models to direct clinical specimens.

The cellular milieu of malignant EOC ascites comprises reticulocytes, lymphoid cells, reactive mesothelial cells, and fibroblasts [18]. The proportions and total amounts of these cell types, cancer and non-cancer, will be variable among different patients and within an individual patient during disease progression. Therefore, spheroids found in patient ascites will likely consist of carcinoma cells as well as some of these other supportive cells of the tumour microenvironment. Figure 2 demonstrates some examples where EOC cells have been co-cultured with fibroblasts to evaluate cell heterogeneity within a spheroid, and whether these different non-cancer cell types have enhanced or reduced capacities for spheroid formation [7]. In addition, EOC spheroids can be co-cultured with immune cell types or mixed populations using peripheral blood mononuclear cells to address immune cell activation or suppression and how that may impact malignant cell survival [76]. In the age of novel immune-oncology strategies, this in vitro co-culture system could prove quite useful to improve the relatively poor performance of these immune-based therapies in the setting of advanced EOC.

Additionally, PDOs can be used in this same co-culture capacity where they include other cell types found in the native TME or cancer-associated stroma. One study conducted a short-term co-culture PDO model by culturing the biopsied material as organoids for only 96 h to maintain these other resident TME cell types that are usually lost after continued passaging [77]. With this short-term PDO system, they investigated immune cell heterogeneity in the TME and observed high levels of CD8 + T cells and NK cells, and mechanisms that may influence tumour cell biology and disease progression. To study other TME factors, Qian and colleagues cultured PDOs with cancer-associated mesothelial conditioned media to demonstrate how secreted elements play a role in cancer cell chemoresistance and stemness [78]. Thus, further development of organoid co-culture models has the potential to address many pathobiological implications of the native TME in an experimentally-tractable system.

In summary, the complex nature of EOC needs to be recapitulated easily, accurately, and reproducibly in vitro to understand the interactions of all cell types within tumours and determine how they influence tumour growth, metastasis and therapy response. Many groups have established that co-culture model systems are integral to gaining better insights of EOC. Adding this extra layer of complexity using spheroids and organoids could eliminate the long and tedious process of developing entirely new model systems for investigating complex cell-cell interactions in EOC pathobiology.

## Future perspectives

**Fig. 3:**
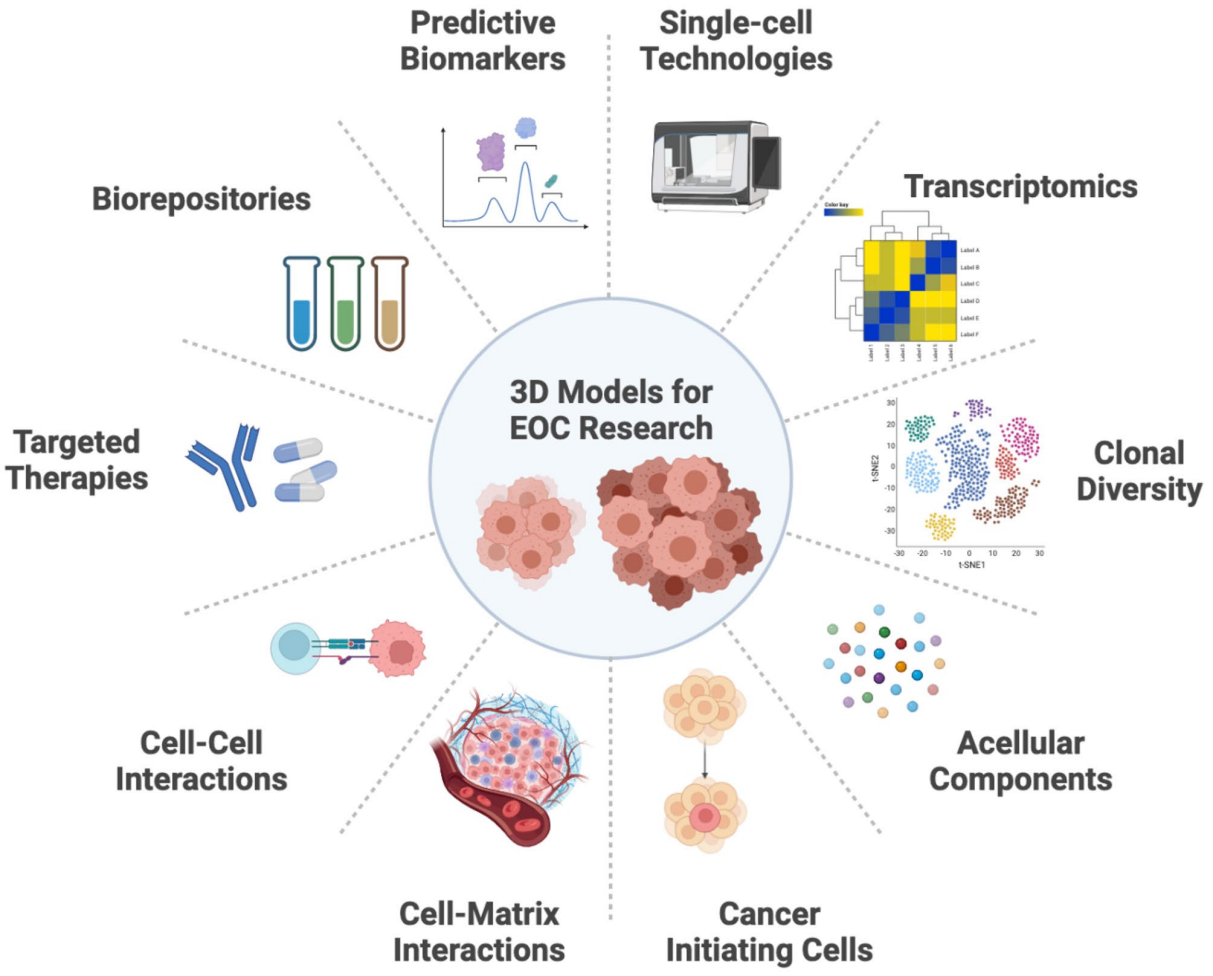
**The applications of 3D spheroid, organoid and co-culture models for EOC research.** Each model system can be used for a multitude of different assays or technologies to further expand our understanding of this disease. Single-cell techniques for transcriptomics or clonal evolution will be popular to determine the diverse nature of EOC. As well, co-culture models can be used to study tumours and spheroids in terms of the acellular components, cancer initiating cells, cell-matrix and cell-cell interactions. These approaches will in turn help researchers discover new targeted therapies, develop biorepositories for future research and potential predictive biomarkers for EOC patients. Created with Biorender.com

After decades of research in EOC, it is clear there are cell signaling pathways and implicated proteins that contribute to cellular dormancy and drug resistance. Metastatic EOC cells are capable of transitioning from this dormant state back into a proliferative or active state when they reach a favourable environment for secondary tumour growth in the peritoneum as illustrated in Fig. 1. However, this entire biological switching process, including specific cell signals and contributing TME factors, is still being elucidated. Therefore, further research needs to be performed to reveal a more complete picture of the dormant-to-proliferative transition and its potential impact on chemoresistance and recurrence. We argue that this can be done best using the various 3D models described in this review (Fig. 2). However, there are still many ways in which we can further develop these models to extend research capacity and incorporate multiple new technologies as they emerge (Fig. 3).

The EOC field has seen only a small number of targeted therapeutics that have succeeded in making it to clinical use (e.g., bevacizumab and PARP inhibitors), yet there is an on-going need to develop other drugs based upon molecular and biochemical vulnerabilities of specific EOC histotypes. EOC spheroids, 3D co-culture organotypic metastasis assays, and now with patient-derived organoids, together they may provide a wealth of new opportunities for discovery and preclinical testing in a more disease-relevant fashion. Since the underlying biology may differ between in situ tumour and mobile spheroid states during disease progression, this indicates varying therapeutic responses within the same patient. PDOs can represent the most recent approach to compare predictive biomarkers expression with immediate responses to established and novel therapeutics [43, 79–81]. Taken together, we propose that the parallel study of both spheroid and organoid models can be used to test personalized medicine approaches targeting different pathobiological states for advanced EOC.

Numerous studies have implicated the tremendous capacity for EOC to evolve during disease progression, particularly in the face of cytotoxic chemotherapy, which essentially drives recurrence of chemoresistance disease. One limitation of these 3D model systems is that long-term culture provides opportunities for clonal selection over time. Hence, short-term spheroid and organoid cultures of patient-derived samples should be considered to prevent creating a divergent and homogeneous population for downstream analyses [56]. However, monitoring of clonal diversity and subsequent evolution observed by genomic sequencing and transcriptome analysis of primary tumours and multiple metastases within individual patients can be directly compared with 3D culture models to study underlying driving mechanisms [82]. Additionally, direct tracking of genetic changes and evolution from FTE to STIC to HGSC, followed by the selective pressures driven by chemotherapy can be readily performed using these 3D models [83]. This represents a viable avenue of future research bridging the gap between precursor lesions and malignant tumours by interrogating molecular and genetic mechanisms in real-time.

This concept can also be connected to the idea of preserving the cellular and molecular heterogeneity seen within the EOC tumour microenvironment of patients. Thus, it is crucial to accurately and efficiently maintain or build this complexity back into 3D culture models. Single cell technologies and spatial transcriptomics could be integral to monitor varying subpopulations of cells and their interactions within precursor STIC lesions and tumours [83–86]. Perhaps single cell analysis of spheroids and organoids will help to resolve the idea of resident ovarian cancer initiating cells. This could be particularly relevant for PDOs to evaluate mechanisms driving propagation and maintenance of heterogeneity within tumours using this tractable 3D ex vivo culture system [82] [83].

Building cellular complexity into 3D cell culture models of EOC tumours is one idea, but perhaps we must also consider the contribution of acellular components within the TME and malignant ascites. For example, complete ascites, or stepwise addition of specific factors present in ascites (e.g., TGFβ1, inflammatory cytokines) could be added directly to 3D cultures to evaluate impact on spheroid formation, cell viability, and even EOC cell-TME interactions [73].

One of the major advantages of in vitro and ex vivo 3D culture models is the ability to perform real-time measurements in an experimentally manipulable and relatively high-throughput way. However, being able to expand in vitro findings to animal models (e.g., patient-derived xenografts) is still crucial for translational research. By developing spheroids and organoids that can be efficiently applied using a complementary animal model to recapitulate mechanistic findings should be a worthy goal for translational EOC research [49, 57, 87].

One on-going limitation in EOC research in a broader sense is the lack of resources and models available to study rare EOC tumour histotypes. Due to its much higher prevalence, protocols are well-established and successful for HGSC, but this is certainly not the case for the other histotypes. To address this, a dedicated objective for wider sharing of resources of PDOs among research programs and biorepositories is needed to successfully pursue novel therapeutics directed against non-HGSC ovarian cancers (e.g., MEK-inhibitors for *KRAS*/*BRAF*-mutated low-grade serous; synthetic-lethal approaches for *ARID1A*-deficient ovarian clear cell cancers) [46].

## Conclusions

Researchers have been incredibly innovative in their use of 3D models for EOC studies. This is evident through the many discoveries of factors controlling spheroid formation that are implicated cell survival, but gaps in our knowledge of the unique nature of EOC pathobiology remain. Thus, we need to continue to advance these 3D models to fully understand tumour dormancy and chemoresistance mechanisms, with a particular emphasis on clonal subpopulations and heterogeneity, to identify more efficacious therapeutic options for future EOC patients. By advancing and improving these models, we have a better chance to help future EOC patients maintain remission and survive this devastating disease.

## Data Availability

Not applicable.

## Abbreviations

4EBP1: eukaryotic translation initiation factor 4E-binding protein 1
AIC: autophagy initiation complex
AKT: protein kinase B
AKT1/2/3: AKT serine/threonine kinase 1/2/3
ALDH1: aldehyde dehydrogenase 1
AMPK: AMP-activated protein kinase
ARID1A: AT-rich interactive domain-containing protein 1A
ARHI: aplasia Ras homolog member I
BME: basement membrane extract
BMP2: bone morphogenetic protein 2
BRCA1/2: breast cancer 1/2
CAMKKβ: calcium/calmodulin-dependent kinase kinase beta
CD133: cluster of differentiation 133/prominin-1
ECM: extracellular matrix
EGF: epidermal growth factor
ER: estrogen receptor
FIGO: International Federation of Obstetrics and Gynaecology
FTE: fallopian tube epithelium
HER2: human epidermal growth factor receptor 2
HGSC: high-grade serous carcinoma
HNF1B: hepatocyte nuclear factor-1 beta
iPSC: induced pluripotent stem cell
LKB1: liver kinase B1
MEK: mitogen-activated protein kinase kinase
mTOR: mammalian target of rapamycin
NK: natural killer
NUAK1: NUAK family kinase 1
PARP: poly adenosine diphosphate-ribose polymerase
PAX8: paired-box 8
PDO: patient-derived organoid
PIK3CA: phosphatidylinositol-4,5-bisphosphate 3-kinase catalytic subunit alpha
PI3K: phosphatidylinositol-3-kinase
PTEN: phosphatase and tensin homolog
PR: progesterone receptor
p70S6K: ribosomal protein S6 kinase beta-1
Rb1: RB transcriptional corepressor 1
Skp2: s-phase kinase associated protein 2
ULK1: unc-51-like kinase 1
Wnt: wingless/integrated
Wnt3a: Wnt family member 3a
ZEB1/2: zinc finger E-Box binding homeobox 1
